# Supplementary material for: Evaluation of the Occurrence of Staphylococcaceae with Reduced Susceptibility to Cefoxitin in Wild Ungulates in Brandenburg, Germany, Based on Land Use-Related Factors
Source: Microbiol Spectr. 2022 Sep 28;10(5):e02560-22. doi: 10.1128/spectrum.02560-22 (PMC9603044; doi:10.1128/spectrum.02560-22)
Supplement: Supplemental file 1 — Table S1. Download spectrum.02560-22-s0001.pdf, PDF file, 0.4 MB [file spectrum.02560-22-s0001.pdf]

Evaluation of the occurrence of *Staphylococcaceae* showing reduced susceptibility to cefoxitin in wild ungulates in Brandenburg, Germany, based on land use-related factors

Rafael H. Mateus-Vargas[a,*], Tobias Lienen[b], Denny Maaz[a], Martin Richter[b], Sven Maurischat[b], Julia Steinhoff-Wagner[a,c]

[a]German Federal Institute for Risk Assessment (BfR), Department Safety in the Food Chain, Berlin, Germany

[b]German Federal Institute for Risk Assessment (BfR), Department Biological Safety, Berlin, Germany

[c]Technical University of Munich, TUM School of Life Sciences, Munich, Germany

*Corresponding author: rafael.mateus-vargas@bfr.bund.de

Supplementary Table 1. General information on sampled animals carrying *Staphylococcaceae* showing reduced susceptibility to cefoxitin.

Hunting district	Animal source	Sex[a]	Age classes[b]	Isolate
A	roe deer	m	1	*S. aureus*
C	roe deer	f	1	*Mammaliicoccus* sp.
	roe deer	f	2	*Mammaliicoccus* sp.
D	roe deer	f	2	*Mammaliicoccus* sp.
	wild boar	m	0	*Mammaliicoccus* sp.
F	wild boar	m	2	*Mammaliicoccus* sp.
G	wild boar	m	2	*S. aureus*
I	red deer	f	1	*Mammaliicoccus* sp.
J	fallow deer	m	1	*Mammaliicoccus* sp.
	fallow deer	m	1	*Mammaliicoccus* sp.
	fallow deer	m	1	*Mammaliicoccus* sp.
	fallow deer	f	2	*Mammaliicoccus* sp.
	fallow deer	f	2	*Mammaliicoccus* sp.
	fallow deer	f	2	*Mammaliicoccus* sp.
	fallow deer	f	2	*Mammaliicoccus* sp.
	fallow deer	f	0	*S. aureus*
	fallow deer	f	0	*S. aureus*
	fallow deer	f	0	*S. aureus*
	fallow deer	m	0	*S. aureus*
	fallow deer	m	2	*S. aureus*
	fallow deer	m	0	*S. saprophyticus*
	fallow deer	f	2	*S. saprophyticus*
K	red deer	m	0	*Mammaliicoccus* sp.
	wild boar	f	1	*Mammaliicoccus* sp.
M	red deer	f	2	*Mammaliicoccus* sp.
	red deer	f	2	*S. epidermis*
	roe deer	m	0	*Mammaliicoccus* sp.
	roe deer	f	0	*Mammaliicoccus* sp.
	roe deer	m	1	*Mammaliicoccus* sp.
	roe deer	f	1	*S. aureus*
	wild boar	m	0	*Mammaliicoccus* sp.
	wild boar	f	2	*Mammaliicoccus* sp.
N	roe deer	f	0	*S. aureus*
O	roe deer	f	0	*Mammaliicoccus* sp.
	roe deer	f	1	*Mammaliicoccus* sp.
	roe deer	m	2	*Mammaliicoccus* sp.
	roe deer	m	0	*S. succinus*
Q	fallow deer	f	0	*Mammaliicoccus* sp.
	fallow deer	m	1	*Mammaliicoccus* sp.
	fallow deer	m	1	*Mammaliicoccus* sp.
	fallow deer	m	2	*Mammaliicoccus* sp.
	fallow deer	f	2	*Mammaliicoccus* sp.
	red deer	f	1	*S. aureus*
	wild boar	f	0	*Mammaliicoccus* sp.
S	roe deer	m	2	*S. saprophyticus*

[a] f = female; m = male

[b] 0 = less than one year old; 1 = between one and two years old; 2 = older than two years
